# Antiproliferative Evaluation of Isofuranodiene on Breast and Prostate Cancer Cell Lines

**DOI:** 10.1155/2014/264829

**Published:** 2014-05-22

**Authors:** Michela Buccioni, Diego Dal Ben, Catia Lambertucci, Filippo Maggi, Fabrizio Papa, Ajiroghene Thomas, Claudia Santinelli, Gabriella Marucci

**Affiliations:** School of Pharmacy, Medicinal Chemistry Unit, University of Camerino, Via S. Agostino 1 62032 Camerino, Italy

## Abstract

The anticancer activity of isofuranodiene, extracted from *Smyrnium olusatrum*, was evaluated in human breast adenocarcinomas MDA-MB 231 and BT 474, and Caucasian prostate adenocarcinoma PC 3 cell lines by MTS assay. MTS assay showed a dose-dependent growth inhibition in the tumor cell lines after isofuranodiene treatment. The best antiproliferative activity of the isofuranodiene was found on PC 3 cells with an IC_50_ value of 29 *μ*M, which was slightly less than the inhibition against the two breast adenocarcinoma cell lines with IC_50_ values of 59 and 55 *μ*M on MDA-MB 231 and BT 474, respectively. Hoechst 33258 assay was performed in order to study the growth inhibition mechanism in prostate cancer cell line; the results indicate that isofuranodiene induces apoptosis. Overall, the understudy compound has a good anticancer activity especially towards the PC 3. On the contrary, it is less active on Chinese hamster ovary cells (CHO) and human embryonic kidney (HEK 293) appearing as a good candidate as a potential natural anticancer drug with low side effects.

## 1. Introduction

Cancer is the leading cause of death worldwide and its relative importance continues to increase. In 2008, it caused 7.6 million deaths and the incidence of cancer continues to increase with an estimated 13.1 million deaths in 2030 [1]. Moreover, an increasing proportion of cancer patients are acquiring resistance to traditional chemotherapeutic agents. This worrying situation requires the development of treatment strategies. Commercially available anticancer drugs, which can be classified by origin as either chemical synthetic drugs or natural drugs, are derived from organisms or plants [2, 3]. Often, synthetic drugs are the only option for cancer chemotherapy [3–5] and their action is not specific for tumor cells since they kill also normal cells generating severe side effects [6]. Natural antitumor drugs derived from organisms or plants were also proven to be effective and less toxic for cancer therapy [6, 7]. In particular, more than 70% of the approved anticancer drugs in the United States of America (from 1981 to 2010) were from natural origin [8]. Screenings of medicinal plants used traditionally as anticancer remedy have provided pharmaceutical industry with effective cytotoxic drugs.

Nonetheless, also plants which were historically used for other purposes may deserve attention of scientists. This is the case of* Smyrnium olusatrum* L. (Apiaceae), well known as wild celery or Alexanders, representing a pot-herb that was cultivated in gardens for many centuries owing to its culinary properties and afterwards it was superseded by the improved form of celery (*Apium graveolens* L.).

From the whole plant, it is possible to obtain essential oils mainly constituted by furanogermacrane-type sesquiterpenes [9]. Their occurrence can be explained by the fact that these molecules are considered to be precursors of sesquiterpene lactones [10] which are in turn regarded as marker compounds of the genus* Smyrnium* [11]. The parent compound of this class of molecules is isofuranodiene ((5E,9E)-3,6,10-trimethyl-4,7,8,11-tetrahydrocyclodeca[b]furan), CAS Registry Number: 57566-47-9, molecular weight: 216.1514), a thermosensitive molecule which, when subjected to high temperatures, undergoes Cope rearrangement to its corresponding elemane derivative curzerene [12]. Isofuranodiene has been also isolated from leaves of* Chloranthus tianmushanensis*, a traditional Chinese medicine used in the treatment of dermatological disorders [13], and from the coral* Leminda millecra* living in Algoa Bay, South Africa [14]. In a previous investigation, this molecule showed inhibitory effects against the proliferation of human colon carcinoma, glioblastoma, and breast adenocarcinoma cells, while its antioxidant and antimicrobial activity was negligible [15]. Taking into account that isofuranodiene can induce cell death, the* in vitro* antiproliferative effect of the molecule on breast and prostate cancer using MDA-MB 231 and BT 474 breast adenocarcinoma and PC 3 Caucasian prostate adenocarcinoma cells has been investigated in the present work. Moreover, in the PC 3 cells, it has been examined the effect of isofuranodiene on cell apoptosis.

## 2. Materials and Methods

### 2.1. Plant Material and Preparation of Isofuranodiene

**Figure 1:**
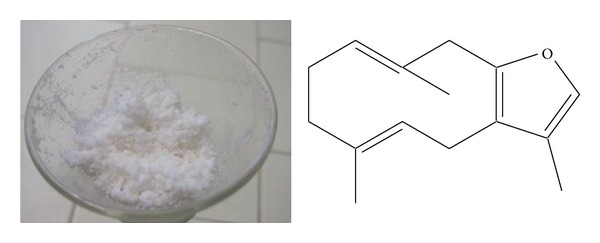
Crystals and chemical structure of isofuranodiene.

**Figure 2:**
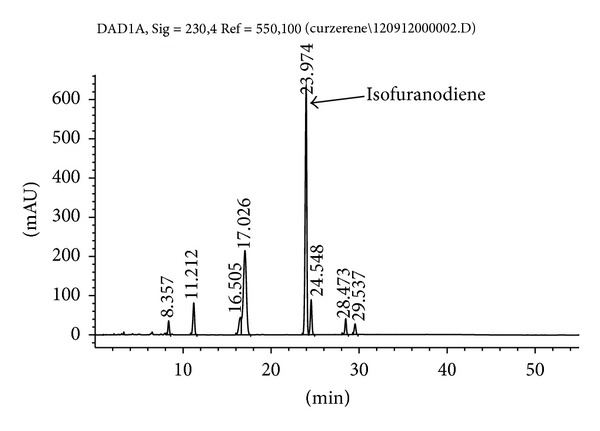
HPLC chromatogram of the essential oil from flowers of* Smyrnium olusatrum*. Isofuranodiene with a retention time of 23.974 min is the main peak (48%). The chromatographic conditions are as follows: column: Kinetex PFP 100A (100 × 4.6 mm i.d., 2.6 *μ*m) from Phenomenex (Torrance, CA); mobile phases: water (A)-acetonitrile (B) (0–15 min: 40% B; 15–30 min: 60% B; 30–40 min 60%B) with a constant flow rate of 1 mL/min; injection volume: 1 *μ*L; detection wavelength: 230 nm.

Isofuranodiene (C_15_H_20_O, crystals, purity 99% as determined by HPLC) (Figure 1) was isolated by crystallization at −20°C from the essential oil of flowers of* Smyrnium olusatrum* L. (Apiaceae) and purified by recrystallization with* n*-hexane. The molecular structure was confirmed by comparison of ^1^H and ^13^C-NMR data obtained on a Varian Mercury plus 400 Spectrometer, using CDCl_3_ as solvent and the solvent signals as internal references, with those reported in the literature [9]. Isofuranodiene was the major constituent, accounting for 48% of the volatile oil (Figure 2). The plant material from which isofuranodiene was isolated was collected in San Giusto (near Pievebovigliana, central Italy, 480 m above sea level, N 43°05′36′′ E  13°08′19′′) in April 2012. The specimen was confirmed by Dr. Maggi using the available literature [16]; hence, it is deposited in the* Herbarium Universitatis Camerinensis* (included in the online edition of* Index Herbariorum* by the New York Botanical Garden: http://sweetgum.nybg.org/ih/) of School of Biosciences and Veterinary Medicine (University of Camerino, Italy) under the accession codex CAME 25675; it is also archived and published in the* anArchive* system (http://www.anarchive.it).

### 2.2. Isofuranodiene Antiproliferative Activity* In Vitro* Studies

#### 2.2.1. Cell Culture

Two human breast adenocarcinomas, MDA-MB 231 and BT 474, and Caucasian prostate adenocarcinoma PC 3 cell lines, in comparison with Chinese hamster ovary (CHO) and human embryonic kidney (HEK 293) cells, were used to study isofuranodiene antiproliferative activity. Human breast adenocarcinoma cell lines MDA-MB 231 and BT 474 were grown adherently and maintained in Dulbecco's Modified Eagle's Medium supplemented with 100 U/mL penicillin, 100 µg/mL streptomycin, and 10% fetal bovine serum (FBS). Caucasian prostate adenocarcinoma cell line PC 3 was grown adherently and maintained in minimum essential medium supplemented with 100 U/mL penicillin, 100 *μ*g/mL streptomycin, and 10% fetal bovine serum (FBS). CHO cells were grown adherently and maintained in Dulbecco's Modified Eagle's Medium high glucose supplemented with 10% FBS, 100 U/mL penicillin, 100 *μ*g/mL streptomycin, 2.5 *μ*g/mL amphotericin, and 2 mM L-glutamine [17]. HEK 293 cells were grown adherently and maintained in the same grow media of CHO with 1 mM sodium pyruvate [18]. All cell lines were cultured at 37°C and aerated with 5% CO_2_ : 95% O_2_.

#### 2.2.2. Evaluation of Antiproliferative Activity

Tested compounds were dissolved in methanol (MeOH) at a concentration of 10.000 *μ*M and diluted with specific cells understudy medium prior to use. Ten thousand cells of each cell line were suspended in 98 *μ*L of specific medium and incubated in a 96-well plate for overnight. After the incubation, 2 *μ*L of the compound was added to the well with the final concentrations of 10–150 *μ*M. After 72 h incubation at 37°C, viability of the cells was determined by 3-(4,5-dimethylthiazol-2-yl)-5-(3-carboxymethoxyphenyl)-2-(4-sulfenyl)-2H-tetrazolium (MTS) assay using Cell Titer 96 Aqueous One Solution Cell Proliferation Assay (Promega Italia Srl) [19].

After the addiction of MTS, in combination with the electron coupling agent phenazine methosulfate, the cells were allowed to incubate for 1 h and absorbance was measured at 492 nm in a microplate reader, GeniosPro. Cell viability was calculated as a percentage using the formula: (mean OD of treated cells/mean OD of control cells) × 100. Results are expressed as percent of control cells which are not treated. The growth control (GC) and growth control with MeOH (GCM) were run for each set of cell line. For cell counting, MDA-MB 231, BT 474, and PC 3 were seeded on to 24-well plates at a density of 7 × 10^4^ cells per well. The cells were treated with different concentrations of isofuranodiene (10–150 *μ*M) for 72 h. After the treatment, the cells were harvested, counted, and compared with GCM. The living cell population was estimated by Trypan blue dye exclusion test. In order to evaluate the kinetics of isofuranodiene, the cells were exposed at different incubation times (6, 12, 24, 48, and 72 h). Recovery experiments were performed by treating cells for 6, 12, 24, 48, and 72 h with the compound and assessing cell proliferation after the washout of the drug up to reach 72 h. All experiments were done in triplicate. The results are expressed as IC_50_, the concentration that produce the 50% inhibition of cell viability.

#### 2.2.3. Morphologic Analysis

To observe PC 3 cells undergoing apoptosis, Hoechst 33258 staining was performed as described by Ghavami et al. [20]. Briefly, 3.5 × 10^5^ cells were grown in each well of a 6-well plate and allowed to adhere. After treatment with 29 *μ*M isofuranodiene for 24 h, the cells were fixed with 4% paraformaldehyde for 30 min at room temperature and then washed twice with PBS. Hoechst 33258 (1 *μ*g/mL) was added to the fixed cells, incubated for 1 h at 37°C in dark, and then washed twice with PBS. Cells were counted and examined by fluorescence microscopy. Apoptotic cells were identified by their characteristic nuclei condensation and fragmentation, whereas nuclei from normal cells demonstrated a normal uniform chromatin pattern. The percentage of apoptotic cells was calculated from the ratio of apoptotic cells to total cells counted.

### 2.3. Statistical Analysis

The data are expressed as mean ± SD from at least three independent experiments. Student's* t*-test was used for statistical analysis. The IC_50_ values were determined by regression analysis after plotting a graph of % cell viability versus drug concentration. Results are considered statistically significant at *P* < 0.05.

## 3. Results and Discussion

**Figure 3:**
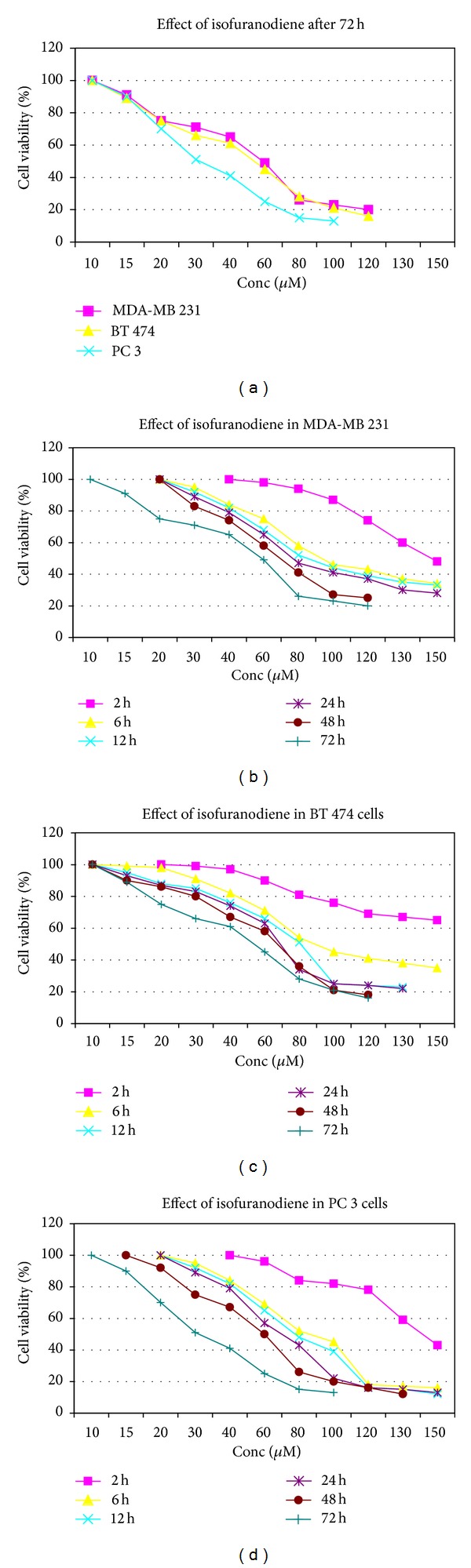
Cell viability in MDA-MB 231, BT  474, and PC 3 cell lines. (a) Percentage of cell viability after treatment with isofuranodiene at doses of 10–150 *μ*M after 72 h of incubation. (b), (c), and (d) Percentage of cell viability after treatment with isofuranodiene at doses of 10–150 *μ*M at different incubation times (2, 6, 12, 24, 48, and 72 h). The results are reported as (viability of treated cells)/(viability of control cells) × 100 and represent the average of three independent experiments with a maximum SD lower than ±8.0.

Human breast adenocarcinomas MDA-MB 231 and BT 474, Caucasian prostate adenocarcinoma PC 3, and two nontumorigenic, CHO and HEK 293, cell lines were treated with various concentrations (10–150 *μ*M) of isofuranodiene for up to three days and the effect on cell viability was examined in comparison with growth control in MeOH (GCM). Cisplatin [cis-diamminedichloroplatinum (II)], an age old anticancer drug, was used as positive control [21]. The % of cell viability of GCM, in comparison with the growth control (GC), was 100% ± 0.65 indicating that the drug solvent (MeOH) does not interfere with the cell viability. In contrast, isofuranodiene provided a good* in vitro* antiproliferative activity against the tested cancer cell lines and a lower cytotoxicity against the two nontumorigenic cell lines (CHO and HEK 293). MTS assay showed a dose-dependent growth inhibition in the tumor cells after isofuranodiene treatment. As it is observed in Figure 3(a), growth inhibition effect of isofuranodiene in MDA-MB 231 cells, after 72 h of incubation, started at 15 *μ*M and increased up to 120 *μ*M (cell viability from 91 ± 4.10% at 15 *μ*M to 20 ± 2.45% at 120 *μ*M versus control 100%, resp.; *P* < 0,05). The effective isofuranodiene concentration for 50% inhibition (IC_50_) in these cells was 59 *μ*M. As shown in Figure 3(a), isofuranodiene induced a reduction also in the BT 474 cell viability (89 ± 5.20% at 15 *μ*M to 19 ± 8.05% at 120 *μ*M versus control 100%, resp.; *P* < 0.05) showing an IC_50_ of 55 *μ*M. In addition, isofuranodiene induced a significant inhibition of PC 3 cell proliferation (90 ± 5. 10% at 15 *μ*M to 13 ± 1.24% at 100 *μ*M versus control 100%, resp.; *P* < 0.05) with an IC_50_ of 29 *μ*M (Figure 3(a)). The IC_50_ values of isofuranodiene are reported in Table 1. Data show that the IC_50_ values of isofuranodiene are comparable to those of the positive control cisplatin (MDA-MB 231 59 versus 39 *μ*M, BT 474 55 versus 37 *μ*M, and PC 3 29 versus 12 *μ*M). Results indicate that the molecule shows antiproliferative activity against all the three cell lines suggesting that isofuranodiene could be considered an anticancer agent such as cisplatin. Notably the major effect is exerted against the PC3 prostate adenocarcinoma cells.

Moreover, data reported in Figures 3(b), 3(c), and 3(d) show the rapid onset of the compound, and, in general, the isofuranodiene induced 50% of cell death in a range of 60–90 *μ*M after 12–24 h. This means that the isofuranodiene is able to induce cell death quite quickly. In addition, when isofuranodiene was added at CHO and HEK 293 cells, the cytotoxicity observed after 72 h was fairly low, IC_50_ of 125 *μ*M and 130 *μ*M, respectively (Table 1). The concentration of 60 *μ*M of isofuranodiene, which in turn provided generally the 50% of antiproliferative activity in breast cancer cell lines, produced only 20% of inhibition in CHO and HEK 293 cell viability, even if the maximum concentration, 150 *μ*M, produced an inhibition of 70 ± 4.5% and 75 ± 3.6% cell viability, respectively. The isofuranodiene antiproliferative effect difference observed at IC_50_ concentrations (30 and 60 *μ*M in PC 3 and MDA-MB 231, BT 474 cells, resp.) among the tumor cell lines and the two noncancer cell lines is significant. This suggests that since the toxicity of the isofuranodiene is being less in noncancer cells it could be a potential anticancer drug with low side effects.

**Figure 4:**
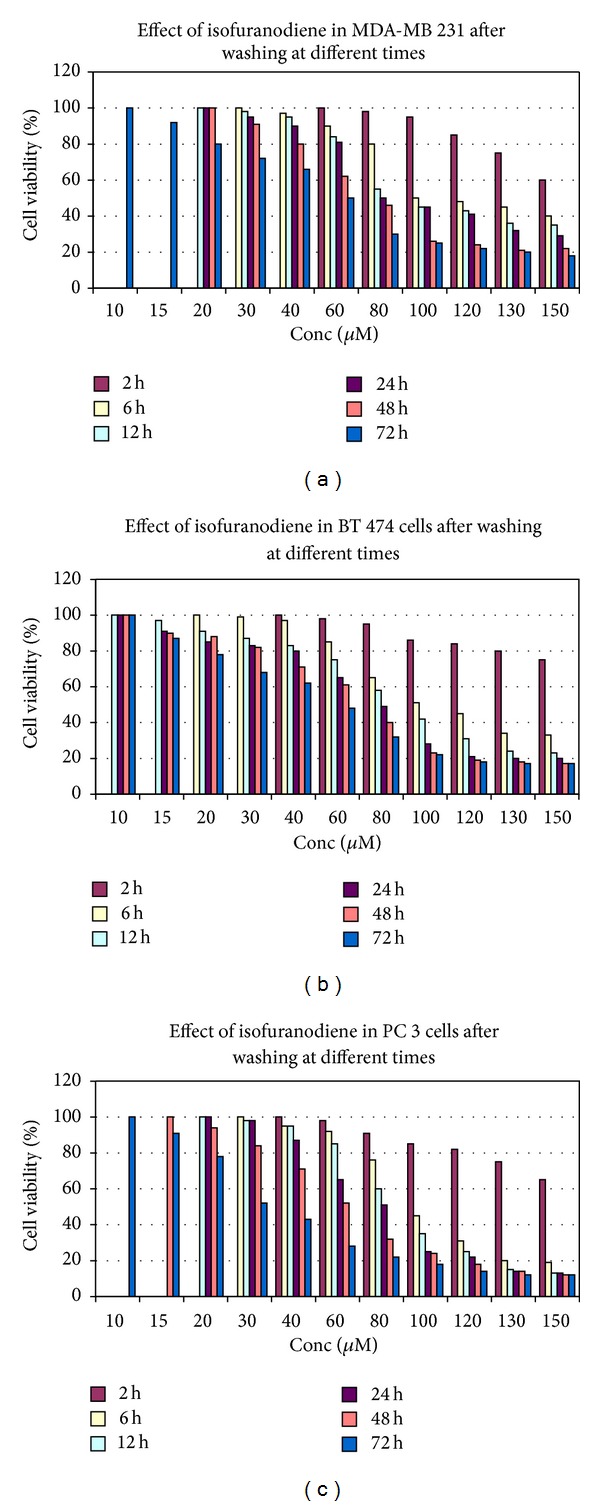
Cell viability assay in MDA-MB 231 (a), BT  474 (b), and PC 3 (c) cell lines expressed in percentage of cell survival after treatment with isofuranodiene at doses of 10–150 *μ*M and at different incubation times (2, 6, 12, 24, 48, and 72 h). After incubation, isofuranodiene was washed out with PBS, and fresh drug-free medium was added. Analysis was performed after 72 h in all the cell lines; results are reported as (viability of treated cells)/(viability of control cells) × 100 and represent the average of three independent experiments with a maximum SD lower than ±8.0.

In order to understand the kinetic of the compound and the reversibility of the antiproliferative effect, the isofuranodiene was evaluated in drug washout experiments. For this purpose, the human cancer cell lines were treated with various concentrations (10–150 *μ*M) of isofuranodiene at different incubation times (6, 12, 24, 48, and 72). Cells were then subsequently washed with buffer (PBS), fresh drug-free medium was added, and residual inhibitory activity was evaluated at 72 h from incubation. As shown in Figures 4(a), 4(b), and 4(c), the data obtained led to the following conclusions: (1) the antiproliferative effect of isofuranodiene increases with the increase of its concentration (10–150 *μ*M) and with time of treatment up to 72 h; (2) after washing, the inhibitory activity is partially reversed after low incubation times but not after 48 and 72 h; (3) in general, the dose that produced the 50% of antiproliferative activity, after washing, is shifted approximately to 80–100 *μ*M except for 2 h, in which the antiproliferative effect is very low, and 48–72 h in which the IC_50_ is very similar before and after washing.

Since antiproliferative effect of isofuranodiene in PC 3 cell line was noteworthy, an additional study was carried out to explore the cell growth inhibition mechanism. For this reason, the Hoechst 33258 assay was performed in order to investigate whether the compound induced cell growth inhibition by cell apoptosis. In this experiment, PC 3 cells were treated with 29 *μ*M (IC_50_) of isofuranodiene for 24 h, and apoptotic cell death was analyzed by Hoechst 33258 staining and quantified by using fluorescence microscopy.

**Figure 5:**
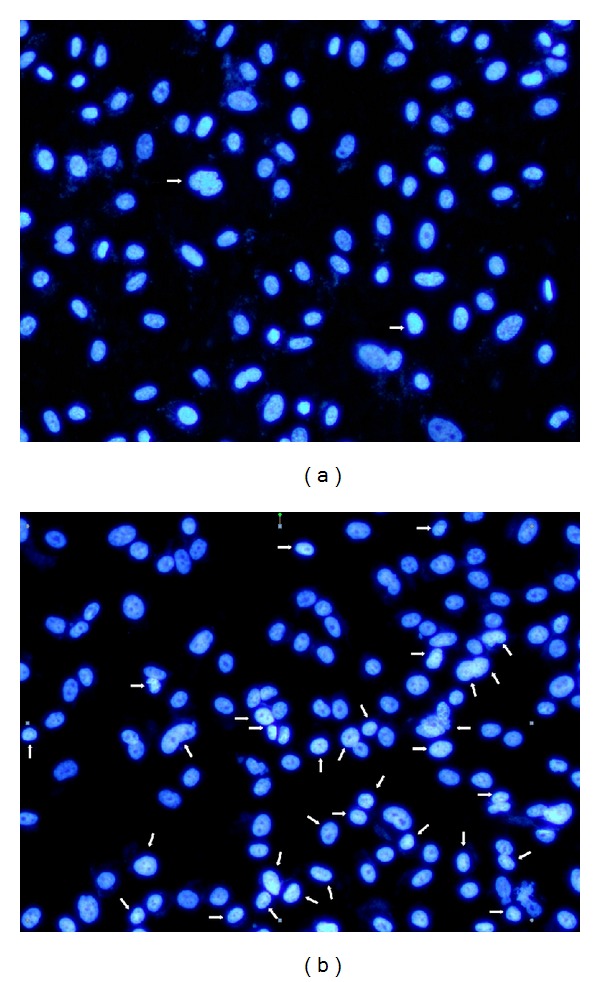
Morphological changes in the nuclei of PC 3 cells. (a) PC 3 control: the majority of cells had uniformly stained nuclei after staining with Hoechst. (b) PC 3 cells after 24 h exposure to 29 *μ*M isofuranodiene induced morphological changes typical of apoptosis.

In the control, most cells contained intact genomic DNA (Figure 5(a)); however, in isofuranodiene-treated cells, many cells had condensed chromatin (Figure 5(b)). Approximately 35.5% of isofuranodiene-treated cells showed DNA changes, but, in the controls, only 1.8% of cells were apoptotic. This significant difference between the control and isofuranodiene-treated cells (Figures 5(a) and 5(b); *P* < 0.05) suggests that isofuranodiene is able to induce apoptosis in prostate cancer cell lines.

## 4. Conclusions

The antiproliferative effect of isofuranodiene at MDA-MB 231, BT 474, and PC 3 cells is comparable to that of cisplatin. This effect is dose dependent increasing with the isofuranodiene concentrations. The best antiproliferative activity was exhibited on PC 3 prostate cancer cells, where the death is induced with an apoptotic mechanism. The lower cytotoxicity against the two nontumorigenic CHO and HEK 293 cells with respect to the cancer ones allows to hypothesize that isofuranodiene could be an anticancer agent endowed with low side effects.

## Figures and Tables

**Table 1:** Antiproliferative activity of isofuranodiene and cisplatin after 72 h.

	Cisplatin IC_50_ [*μ*M]^a^	Isofuranodiene IC_50_ [*μ*M]^a^
Cell line		
MDA-MB 231	39 ± 1.05	59 ± 2.36
BT 474	37 ± 1.65	55 ± 2.54
PC 3	12 ± 0.98	29 ± 1.85
CHO	—	125 ± 3.94
HEK 293	—	130 ± 2.98

^a^Concentration of compound required for 50% inhibition of cell viability determined using MTS assay. Cells were treated with concentrations ranging from 10 to 150 *μ*M for 72 h. The results are reported as the average of three independent experiments.

## References

[1] Soerjomataram I, Lortet-Tieulent J, Parkin DM (2012). Global burden of cancer in 2008: a systematic analysis of disability-adjusted life-years in 12 world regions. *The Lancet*.

[2] Chang C-C, Chen W-C, Ho T-F, Wu H-S, Wei Y-H (2011). Development of natural anti-tumor drugs by microorganisms. *Journal of Bioscience and Bioengineering*.

[3] Ma X, Wang Z (2009). Anticancer drug discovery in the future: an evolutionary perspective. *Drug Discovery Today*.

[4] DeVita VT, Chu E (2008). A history of cancer chemotherapy. *Cancer Research*.

[5] Chabner BA, Roberts TG (2005). Chemotherapy and the war on cancer. *Nature Reviews Cancer*.

[6] Cragg GM, Grothaus PG, Newman DJ (2009). Impact of natural products on developing new anti-cancer agents. *Chemical Reviews*.

[7] Ravelo ÁG, Estévez-Braun A, Chávez-Orellana H, Pérez-Sacau E, Mesa-Siverio D (2004). Recent studies on natural products as anticancer agents. *Current Topics in Medicinal Chemistry*.

[8] Newman DJ, Cragg GM (2012). Natural products as sources of new drugs over the 30 years from 1981 to 2010. *Journal of Natural Products*.

[9] Maggi F, Barboni L, Papa F (2012). A forgotten vegetable (*Smyrnium olusatrum* L., Apiaceae) as a rich source of isofuranodiene. *Food Chemistry*.

[10] Kawabata J, Fukushi Y, Tahara S, Mizutani J (1985). Isolation and structural elucidation of four sesquiterpenes from *Chloranthus japonicus* (Chloranthaceae). *Agricultural and Biological Chemistry*.

[11] El-Gamal AA (2001). Sesquiterpene lactones from *Smyrnium olusatrum*. *Phytochemistry*.

[12] Setzer WN (2008). *Ab initio* analysis of the Cope rearrangement of germacrane sesquiterpenoids. *Journal of Molecular Modeling*.

[13] Wu B, Chen J, Qu H, Cheng Y (2008). Complex sesquiterpenoids with tyrosinase inhibitory activity from the leaves of *Chloranthus tianmushanensis*. *Journal of Natural Products*.

[14] McPhail KL, Davies-Coleman MT, Starmer J (2001). Sequestered chemistry of the Arminacean nudibranch *Leminda millecra* in Algoa Bay, South Africa. *Journal of Natural Products*.

[15] Quassinti L, Bramucci M, Lupidi G (2013). *In vitro* biological activity of essential oils and isolated furanosesquiterpenes from the neglected vegetable *Smyrnium olusatrum* L. (Apiaceae). *Food Chemistry*.

[16] Pignatti S (1982). *Flora d’Italia*.

[17] Volpini R, Buccioni M, Dal Ben D (2009). Synthesis and biological evaluation of 2-alkynyl-N6-methyl-5′-N-methylcarboxamidoadenosine derivatives as potent and highly selective agonists for the human adenosine A3 receptor. *Journal of Medicinal Chemistry*.

[18] Buccioni M, Marucci G, Ben DD (2011). Innovative functional cAMP assay for studying G protein-coupled receptors: application to the pharmacological characterization of GPR17. *Purinergic Signalling*.

[19] Riss TL, Moravec RA (1992). Comparison of MTT, XTT and a novel tetrazolium compound MTS for *in vitro* proliferation and chemosensitivity assays. *Molecular Biology of the Cell*.

[20] Ghavami S, Kerkhoff C, Los M, Hashemi M, Sorg C, Karami-Tehrani F (2004). Mechanism of apoptosis induced by S100A8/A9 in colon cancer cell lines: the role of ROS and the effect of metal ions. *Journal of Leukocyte Biology*.

[21] Macciò A, Madeddu C (2013). Cisplatin: an old drug with a newfound efficacy—from mechanisms of action to cytotoxicity. *Expert Opin Pharmacother*.

